# The magnitude of common mental illness and associated factors among adult holy water users in Amhara regional state Orthodox Tewahido churches, Ethiopia, 2021

**DOI:** 10.1186/s12888-023-04524-2

**Published:** 2023-01-19

**Authors:** Amsalu Belete, Moges Wubneh Abate, Adane Birhanu Nigat, Agimasie Tigabu, Berihun Bantie, Gebrie Kassaw Yirga, Chalie Marew Tiruneh, Tigabu Desie Emiru, Nigusie Selomon Tibebu, Getasew Legas, Sintayehu Asnakew, Belete Gelaw Walle, Moges Yinges Yitayew, Simegnew Kibret, Kassa Abebaw Tesema

**Affiliations:** 1 Department of Psychiatry, College of Health Sciences, Debre Tabor University, P.O.Box:272, Debre Tabor, Ethiopia; 2 Department of Adult Health Nursing, Debre Tabor University, P.O.Box:272, Debre Tabor, Ethiopia; 3 Department of Pediatrics and Child Health Nursing, College of Health Sciences, Debre Tabor University, P.O.Box:272, Debre Tabor, Ethiopia; 4 Department of Pediatrics and Child Health Nursing, College of Health Sciences, Wolayta Sodo University, Wolayta, Ethiopia; 5 Ethiopian Red Cross Society, South Gondar Branch, Debre Tabor, Ethiopia; 6 Department of Anesthesia, College of Health Sciences, Debre Tabor University, P.O.Box:272, Debre Tabor, Ethiopia; 7 Department of Human Physiology, School of Medicine, Debre Markos University, Debre Tabor, Ethiopia

**Keywords:** Magnitude, Common mental illness, Holy water, Amhara, Ethiopia

## Abstract

**Background:**

Because they are prevalent, persistent, and have substantial negative effects on physical health, psychological well-being, and economic implications, common mental illnesses like depression, anxiety, and somatic complaints are major public health problems. Patients with mental illness are devoted to religious therapy including holy water as a coping mechanism for their illnesses. The aim of this study was to investigate the magnitude and associated factors of common mental illness among adult holy water users.

**Methods:**

Facility-based cross-sectional study design was conducted in Amhara regional state orthodox Tewahido churches. A simple random sampling techinque was used to select participants. Data were collected by using the Brief Psychiatric Rating Scale for mental illnesses symptoms and a structured interviewer administered questionnaire was used. Data were entered into EpiData version 4.6 and exported to SPSS version 25 for analysis. Variables with a *p*-value of 0.25 in the bivariable logistic regression were entered into multivariable logistic regression.

**Result:**

Three hundred eighty-two participants were involved in the study. The magnitude of Common mental illnesses among holy water users was 58.9%. Unemployed, using more than one substance, having Poor and moderate social support, current daily alcohol drinker and past history of mental illness were significantly associated with common mental illness.

**Conclusion:**

The magnitude of common mental illness among adult holy water users was high. Giving special attention to decreasing unemployment, establishing social support services, and decreasing substance utilization are the keys to preventing common mental illnesses.

## Introduction

### Back ground

Mental illnesses are health conditions involving changes in emotion, thinking or behavior or a combination of these which leads to distress and problems of functioning in social, work or family activities [1]. Many people who have a mental illness do not want to talk about it [2]. Mental health conditions are treatable which can affect anyone regardless of age, gender, geography, income, social status, race/ethnicity, religion/spirituality, background or other aspect of cultural identity [3].

Neuropsychiatric disorders account for 13% of the total Disability Adjusted Life Years (DALYs) lost due to all diseases and injuries in the world and were estimated to increase to 15% by the year 2020. Worldwide, five of the ten leading causes of disability and premature death are psychiatric illnesses [4]. According to global burden of diseases 2010 among 291 diseases, injuries, and risks from 187 countries indicated that common mental illness (depressive disorder 41% and anxiety disorder 15%) are the third cause of Disability-adjusted life years (DALYs) next to circulatory and cancer diseases [5].

Common mental illnesses like depression, anxiety, and somatic complaints are global health concerns which have substantial negative effects on physical health, psychological well-being, and economic implications because of high prevalence and chronicity [6]. Recent statistics showed that one in five people experienced common mental illnesses in the past year, and 29.2% of people have experienced episodes of common mental disorders throughout their lives [6]. The overlaps of somatic, anxious and depressive syndromes are frequent in the general population [7].

Various studies showed that the magnitude of common mental illnesses varied across the world. In Great Britain (England, Wales and Scotland) 24.6% of the study participants were suffered from common mental illnesses [8]. Another study conducted in Brazil reported that the magnitude of common mental illnesses was 29.3 %[9]. Evidence conducted in China showed that about 34.4% of the respondants were had common mental illnesses [10]. In Africa, the estimated magnitude of common mental illnesses in Tanzania was 24–28.8 %[11, 12]. The magnitude of common mental illnesses was 10.3% in Kenya and 27% in South Africa respectively [13, 14]. Studies done in Ethiopia showed that the magnitude of common mental illnesses was ranged from 14.9 to 33.6% [15–18].

Mental illnesses have great impact on the economical aspect of the population. It directly costs actual expenditures for the care of the mentally ill by public and private agencies [19]. It has also indirect costs based on the annual loss of production, annual earnings, and work years by patients [20]. In addition, mental health ilnesses meaningfully adds to a cycle of poverty where people who experiences social suffering and poverty are at augmented risk of mental illness, and on the other hand, those with mental illnesses are at amplified the threat of poverty [21].

According to a study conducted in Ethiopia, most people were considered that mental disorders are results from sprites and evil forces. For these reasons they prefer religious institutions than mental treatment for curative practices habitually using holy water sites [22]. Another study done in Ethiopia supported that most Ethiopians blames supernatural forces for mental health issues. As a result, the majority of people were seen turning to supernatural remedies such as prayer, fasting, holy water sprinkles, and meeting with those who were believed to possess special abilities for curing mental health issues [23]. The most popular method of treating mental illnesses in Ethiopia is holy water, which is used by Orthodox Christian who think it has healing properties that embody the spirit of Christ [24]. People of Orthodox Christian religion (43.5% of the population )[25] and the wider public [26] both used holy water because of a widely held cultural and interreligious belief.

Eventhough the burden of common mental illnesses are higher in low and middle-income countries; including Ethiopia, the health services delivery setting (primary health care setting) for clients who are suffering from mental illnesses is extremely limited. So due to these reasons, instead of visiting health care services; most clients are forced to visit holy water sites. So far, no study was conducted to assess the magnitude of common mental disorder and its associated factors among adults holy water user in the study setting. Therefore, this study was intended to assess the magnitudeof common mental illness and its associated factors among adults holy water user. More over, the result will give significant information to policymakers, important for early interventions which help to reduce the magnitude of common mental illness and to improve the clients’ quality of life. It will be used for the researchers as a baseline data.

## Methods

### Study design, period and setting

Institutional based cross-sectional study was conducted from November 28 –December 28, 2021, in Amhara regional state Orthodox Tewahido churches, Ethiopia. The study was conducted in Amhara nationalregional state Orthodox churches, which have holy water users for different purposes like the healing of mental illness. Amhara Region is a regional state in Northwest Ethiopia, and its capital city is Bahir Dar. Iaccording to central statistical agency Ethiopia (CSA), 2017 the total population were 21,134,98 8[27]. The study was conducted in five randomly selected churches, namely Wonkshet Gebreal, Gono Gebreal, Megendi Giorgis, Andasa Giorgis, and Tsadikane Maryam. Wonkshet Gebreal and Gono Gebreal located in South Gondar district, Megendi Giorgis and Andasa Giorgis in west Gojjam district, and Tsadikane Maryam located in North Shewa district.

### Source population

All clients who used Orthodox Tewahido churches holy water in Amhara region.

### Study population

All adults who used Orthodox Tewahido churches holy water in Amhara region during the study period.

### Inclusion criteria and exclusion criteria

People, who use Orthodox Tewahido churches holy water in the Amhara region and age of 18 and beyond were involved in the study. On the other hand individuals who had problem of communication, and holy water user who interrupted the interview were excluded from the study.

### Sample size determination

The single population proportion formula was used to determine the required sample size by considering a 50% estimated proportion of mental illness among holy water user adults, since no study was done on this problem in Ethiopia.

By considering 95% confidence interval (CI) and 5% as margin of error, sample was calculated as follows:

$$\mathrm n\;=\;\left({\mathrm z}_{\mathrm\alpha/2}\right)^2\;\mathrm p\;\left(1-\mathrm p\right)/\mathrm d^2$$

$$\text{n}={(1.96)}^20.5\ast0.5=384.16=384$$

$${(0.5)}^2$$

Then total sample size = 384 + 10% non-response rate (39) = 423.

### Sampling techniques

First, permission was obtained from the selected church administrators. Then, lists of holy water users at selected churches were taken from the registration books. After listing, the selection of participants has conducted by the lottery method. The number of samples in each selected churches was distributed proportionally based on the total number of holy water users in individually selected churches.

### Dependent variable

Common mental illness.

### Independent variable

#### Socio demographic variables and psychosocial factor

Sex, Age, Ethnicity, Religion, Marital status, Educational status, Occupation, Residence, Social support

#### Behavioral factors

Alcohol, Tobacco, and Khat.

#### Clinical factors

Previous mental disorder, Family history of mental illness, known medical/surgical disease.

### Operational definitions

#### Common mental illness

Those participants who had self-report somatic illness, anxiety and depression by using Brief Psychiatric Rating Scale (BPRS) [28, 29].

#### Current and ever use of substance

Using a specific substance for non-medical purpose in the last 3 months and using of a particular substance for non-medical purpose once in lifetime respectively [30].

### Data collection tools

Data collection was conducted by using an interviewer-administered structured questionnaire, interview and observation by Brief Psychiatric Rating Scale (BPRS) for mental illness symptoms. Data were collected by four BSc psychiatric nurses and supervised by two MSc psychiatry nurses. BPRS has 24 items with seven scale measurements ranging from “not present” to “extremely severe, the first three items were used to asess common mental illnesses (somatic concern, anxiety and depression) [29, 30]. The Extent of soscial support was assessed by using three-item Oslo social support scale with a range of between 3 and 14. “scoring of 12–14 = strong social support”, “score of 9–11 = moderate social support and”, “score of 3–8= poor social support” scale [31].

### Data quality assurance

The questionnaire was first prepared in English and translated to Amharic language for data collection and back re-translated to English language by fluent speaker language experts to maintain its concistency. The training was given to data collectors and supervisors on BPRS. The pre-test was done on 5% of holy water users at Recha Gebreal church; 2 weeks before the start of actual data collection and revision was made accordingly. Reliability was checked and Cronbach’s alpha was 0.921. The supervisors and principal investigator have checked the completeness and consistency of the filled questionnaires daily.

### Data processing and analysis

Data were coded, filtered, and entered into EpiData version 4.6 and then exported to SPSS version 25 for analysis. Binary logistic regression analysis was used to determine the association between the dependent and independent variables. Firstly, bivariable logistic regression was performed and all variables with *P* ≤ 0.25 in the bivariable analysis were included in the final model of multivariable analysis in order to control all possible confounders. The goodness of fit was tested by Hosmer-Lemeshow statistic and the data fitted to the model. The direction and strength of statistical association were measured by the odds ratio with 95% CI. The adjusted odds ratio along with 95% CI were estimated to identify factors associated with complications by using multivariable analysis in binary logistic regression. Finally, *P*-value ≤0.05 was considered to declare as a statistically significant.

## Results

### Socio demographic characteristics

Three hundred eighty-two participants were involved in the study with a response rate of 90.3%. Among respondents, 253 (56.3%) were females and about half (47.9%) of them were in the age group of 26–40 with a mean age of 35.69 (SD ± 12.43). Of the total respondents, 184 (48.2%) were separated. Regarding social support conditions, 175 (45.8%) were had poor social support. In relation to occupational status, 66% of them were unemployed (Table 1).

**Table 1:** Socio demographic characteristics and psychosocial factors among adults holy water user in Amhara regional state Orthodox Tewahido churches, Ethiopia, 2021 (*n* = 382)

Variable		Frequency	Percent
Sex	Male	167	43.7
	Female	215	56.3
Age	18–25	50	13.1
	26–40	183	47.9
	41–64	77	20.2
	65^+^	72	18.8
Marital status	Single	86	22.5
	Separated	184	48.2
	Married	112	29.3
Social support	Poor	90	23.6
	Moderate	175	45.8
	Strong	117	30.6
Ethnicity	Amhara	382	100
Placeof residence	Rural	246	64.4
	Urban	136	35.6
Occupational status	Employed	130	34.0
	Unemployed	252	66.0

### Behavioral factors

About 101 (26.4%) of the respondents were used two or more substance in their lifetime (Table 2).

**Table 2:** Behavioral factors among adult holy water users at Amhara regional state Orthodox Tewahido churches, Ethiopia, 2021 (*n* = 382)

Variable	Yes/no	Frequency	%
Use of two or more substances	No	281	73.6
	Yes	101	26.4
Alcohol	Daily	90	23.6
	Weekly	63	16.5
	twice monthly	160	41.9
	Never	69	18.1
Khat	Daily	23	6.0
	Weekly	3	0.8
	Twice monthly	4	1.0
	Never	352	92.1
Tobacco	Daily	6	1.6
	Weekly	6	1.6
	Twice monthly	4	1.0
	Never	366	95.8

*NB* Never: not used in the last 3 months

### Clinical related factors

Of 382 respondents, 194 (50.6%) had a past history of psychiatric illness and 53 (13.9%) had a family history of mental illness. Ninety-two (24.1%) of the study participants had a history of known medical or surgical condition (Table 3).

**Table 3:** Clinical characteristics of adult holy water users at Amhara regional state Orthodox Tewahido churches, Ethiopia, 2021 (*n* = 382)

Variables		Frequency	Percent
Past history of psychiatric illness	No	188	49.2
	Yes	194	50.8
Family history of mental illness	No	329	86.1
	Yes	53	13.9
Known medical or surgical conditions	No	290	75.9
	Yes	92	24.1

### Magnitude of common mental illness

Among 382 study participants, 225(58.9%) had a BPRS score of > 31. Therefore, the magnitude of common mental illness was 58.9% with 95% CI (53.9–63.6).

Among mentally ill individuals, 133 (59.1%) were females and 121 (53.7%) were separated. Of mentally ill individuals, 79 (35.1%) were using more than one substance in their lifetime and 39.1% of them had a previous history of mental illness.

### Factors associated with common mental illness

Being unemployed holy water users were 1.71 times (AOR = 1.71, 95% CI = (1.002–2.92)) more likely to had common mental illness (Table 4).

**Table 4:** Bivariate and multivariable logistic regression mental illness among adult holy water users at Amhara regional state Orthodox Tewahido churches, Ethiopia, 2021 (*n* = 382)

Variables		Common mental illness		COR(95% CI)	AOR(95% CI)	*p*-value
		Yes	No			
Residence	Rural	151	95	1.33 (0.87–2.04)	1.17 (0.70–1.97)	0.552
	Urban	74	62	**1**		
Social support	Poor	63	27	3.24(1.810–5.792)	2.45 (1.22–4.94)	**0.012**
	Moderate	113	62	2.53 (1.564–4.089)	2.65 (1.50–4.66)	**0.001**
	Strong	49	68	**1**	**1**	
Occupational status	Employed	63	67	**1**	**1**	
	Unemployed	162	90	1.91 (1.246–2.942)	1.71 (1.002–2.92)	**0.049**
Ever use of more than one substance	No	150	131	**1**	**1**	
	Yes	75	26	2.52 (1.52–4.17)	**2.24 (1.22–4.13)**	**0.009**
History of mental illness	Yes	129	58	2.29 (1.51–3.48)	2.17 (1.35–3.47)	**0.001**
	No	96	99	**1**		
Marital status	Single	48	38	1.02 (0.578–1.79)	1.56 (0.78–3.12)	0.208
	Separated	115	69	1.34 (0.83–2.12)	1.34 (0.80–2.31)	0.261
	Married	62	50	**1**		
Current alcohol use	Never	25	23	**1**	**1**	
	twice monthly,	58	18	1.68 (0.65–4.34)	1.76 (0.58–5.31)	0.319
	Weekly	122	106	1.02 (0.55–1.89)	1.09 (0.55–2.14)	0.808
	Daily	19	10	2.85 (1.32–6.16)	2.69 (1.15–6.32)	**0.023**
Comorbidity	Yes	60	32	1.42 (0.87–2.31)	0.83 (0.467–1.48)	0.527
	No	165	125	**1**		

1 = constant; Hosmer Lemshow goodness fit = 0.069

*AOR* Adjusted Odds Ratio, *CI* Confidence Interval, *COR* Crude Odds Ratio

## Discussion

The main aim of this study was to assess the magnitude and associated factors of common mental illness among adults holy water user in Amhara regional state Orthodox Tewahido churches, Ethiopia.

The magnitude of common mental illness among adults holy water user in the Amhara region Orthodox Tewahido churches holy water users was 58.9% (95% CI, 53.9–63.6). The finding is higher than the study done in Great Britain 24.6 %[8], Brazil 29.3 %[9], China, Bangladesh 52.5 %[32], 34.4 %[10], South Africa 27 %[13], Kenya 10.3 %[14], and Ethiopia 29.7 %[18]. This difference is mainly due to the study setting. Because our study setting were holy water sites the chance of getting large number of common mental illnesses is high compared with community based setting. This high magnitude of common mental illness can be due to the reason that, mentally illpeople prefer religious places than psychiatric institutions due to their thought, sprit, and possession that supernatural are the cause of mental illnesses [33]. The other reason is misunderstanding of population around the study area that is mental illness is not treatable by psychomedications rather they believed that the illness is due to punishment from God or creatures [18].

This study revealed that common mental illness is more likely in unemployed individuals as compared to those who were employed. This is supported by studies done in Australia [34], South Africa [13], Tanzania [12], and Ethiopia [18, 35]. This can be explained by common mental illnesses among unemployed may result from tension due to the incapability to accomplish economic and social costs. Unemployed is also at risk for substance dependence tributary to desperateness, and difficulties in managing social and familial needs, which all lead to common mental illness.

Holy water users who used more than one substance were more likely to have common mental illnesses as compared to those who do not have a substance use history. This result is supported by the study [36]. This is due to the interactions between mental illnesses and substance use that leads to physiological, economic, and social functioning impairment on an individual [37].

The result in this study showed that those who have a previous history of mental illness were more likely to have common mental illness. The finding is similar with the studies done in Ethiopia [18, 35]. This is due to those who had previous history of psychiatric illnesses either they might not got psychiatric treatmentor not taken their medication properlyand the root cause of illnesses might not be managed; the chance of relapsing is high.

This study revealed that having poor and moderate social supports were more risk for the occurrence of common mental illness. This finding is inline with the studies done in Ethiopia [18, 33]. The possible explanation for this is those individuals who are poor, and moderate social support may exposed to certain harmful things, stressors or substances like alcohol drinking and might be developed or susceptible for common mental illness.

### Limitation of study

The study did not analyze the effect of a specific medical illness and undiagnosed medical illness on common mental illnesses.

## Conclusion

The magnitude of common mental illness among adults holy water user was high. The reasons for this figure reflect that the insight of community towards mental illnesses entirly depends on religious sites. To minimize the magnitude of common mental illness; stakeholders should work together with religious fathers or leaders to bring these patients to mental clinics. Different factors were identifed which contributed for common mental illness. Giving special attention to decreasing unemployment, establishing social support services, decreasing substance utilization by providing substance rehabiltation centers in health services and early management of mental illness helps to decrease common mental illness.

## Data Availability

All data are available with in the manuscript and supplementary files.
